# Playing your pain away: designing a virtual reality physical therapy for children with upper limb motor impairment

**DOI:** 10.1007/s10055-021-00522-5

**Published:** 2021-06-09

**Authors:** Ivan Phelan, Penny Jayne Furness, Maria Matsangidou, Alicia Carrion-Plaza, Heather Dunn, Paul Dimitri, Shirley A. Lindley

**Affiliations:** 1 Centre for Culture, Media and Society, College of Social Sciences and Arts, Sheffield Hallam University, Sheffield, S1 1WB UK; 2 Department of Psychology, Sociology and Politics, College of Social Sciences and Arts, Sheffield Hallam University, Sheffield, S1 1WB UK; 3 Sheffield Children’s NHS Foundation Trust, Sheffield, S10 2TH UK

**Keywords:** Virtual reality, Patient-centred design, Upper limb motor impairment, Pain management, Children’s rehabilitation

## Abstract

Children with upper limb motor impairment often undergo repetitive therapeutic physiotherapy sessions to minimize functional disabilities of the affected area. Even though therapeutic processes can improve functional outcomes and minimize persistent disabilities, patients often neglect to participate fully in physical therapies due to the associated procedural pain. Over recent decades, there has been a growing interest in designing non-pharmacological interventions which aim to minimize pain during physical therapies and improve functional outcomes. Via two interrelated studies, we explored the use of virtual reality (VR) as a tool to provide therapeutic physiotherapy for child patients in an out-patient hospital department. We found that VR is an effective solution for children with upper limb motor impairment undergoing painful therapeutic process within a hospital environment. VR can improve functional disabilities, alleviate perceived pain, reduce the perceived difficulty of rehabilitation exercises, increase exercise duration and produce positive emotions towards the therapy.

*Trial registration number and date of registration* Protocol ID NCT03998995. Release Date: June 25, 2019.

## Introduction

Children who are suffering from Upper Limb Motor Impairment (ULMI) must often undergo repetitive therapeutic physiotherapy sessions to regain movement or minimize functional disabilities of the affected area. Therapeutic physiotherapy sessions usually include upper body movements and exercises such as overhead, side-front, and back arm raises and curls. These physical therapeutic processes are fundamental components of rehabilitation because they improve functional outcomes and minimize persistent disabilities; however, patients often neglect to participate fully in physical therapies due to acute procedural pain (Matsangidou et al. 2017b).

Pain has been characterized as one of the most common medical complaints (Malloy and Milling 2010), however, clinicians encounter difficulties in treating pain due to its complex and subjective nature (Gold et al. 2007; Mahrer and Gold 2009). Pain has been defined as a sensory and emotional experience that causes discomfort to the individual following actual or perceived tissue injury (Merskey and Bogduk 1994). As such, it is both nociceptive and subjective, with the same sensory signal of pain giving rise to different levels of pain intensity among individuals and situations.

In recent decades, computer technology has brought to light new opportunities for pain management in painful therapeutic processes (Chau et al. 2020; Furness et al. 2019; Phelan et al. 2019). Virtual reality (VR) is a representative example of this type of technology since it allows users to experience a computer-simulated reality with visual, auditory, tactile and olfactory interactions (Ma and Zheng 2011) which results in distracting the patient from perceiving nociceptive signals and pain (Matsangidou et al. 2017a, b ). VR for pain management has been introduced in the research community as VR-analgesia and it appears to be an advanced form of analgesia caused by conventional distraction (Hoffman et al. 2004; 2006; Schmitt et al. 2011). Research on neurobiological mechanisms has shown that VR can reduce pain perception by withdrawing the subject's attention from the signals of pain (Gold et al. 2007).

Many studies have found VR to be an efficient and beneficial form of pain relief for children in rehabilitation (Khadra et al. 2018; Parsons et al. 2009). VR games were more promising than conventional training alone to improve limbs’ functional abilities in children with cerebral palsy (Jannink et al. 2008; Sharan et al. 2012). Studies also found that VR training initially has a high intrinsic motivational power that increased children’s level of engagement in and enjoyment of physical activities (Jannink et al. 2008). Other recent works found good acceptability of computer game-based interventions to encourage children's engagement in rehabilitation exercise at home and recommended more entertaining games to increase motivation and compliance in the child (Gerber et al. 2016).

Even though the effectiveness of VR on pain management has been well documented, none of the above papers have provided illustrations in response to the design elements of an effective VR system. We, therefore, believe that through this paper, we shed some light on the design opportunities for the future deployment of VR in healthcare services. This is done through the qualitative assessment of the attitudes of young patients, consultees/ family members (e.g. parents) and clinicians towards this technology. This paper aims to understand whether VR would be accepted by young patients, family members and clinicians and whether the use of this technology could be translated into positive results. We also need to examine the unique features, advantages and limitations of VR so that it can be deployed successfully in larger scale hospital settings. In addition, we look to understand what other benefits VR could provide to this patient group (e.g. apart from eliminating pain), and how we can design VR to enhance and maximize these benefits in the future.

This study describes the development of a VR application for the treatment of children who are suffering from ULMI. The usability and applicability of the application were evaluated through two interrelated studies. Study 1 assessed the VR system’s usability with healthy children to inform the design and Study 2 deployed the system into a children’s hospital to identify the potentials of VR technology in healthcare settings.

## Methods

### Ethics and recruitment

Healthy participants for the pre-clinical developmental consulting study were recruited from local schools. Ethical approval for the pre-clinical study was gained from the University (ER-5456174).

Participants with upper limb impairments, consultees and clinical staff were recruited from Sheffield Children’s NHS Foundation Trust in the UK, which is one of three dedicated children’s hospitals providing integrated healthcare for children and young people. Ethical approvals were gained from the hospital (*SCH: 2178*) as well as the University (*ER-5456174*) and the National Health Service (*IRAS: 243763*) Research Ethics Committees.

All participants (healthy children, children with ULMI, family members and clinicians) signed a consent form prior to the study. The study was performed in accordance with the Declaration of Helsinki. Parental and age-appropriate children's information sheets were developed, following good practice when working with children. To ensure the participants' voluntary participation, parents were asked to discuss the study with their child before giving consent. Ongoing consent was also checked verbally with the child before and during procedures.

### Sample, study design and procedure

The study design was developed in consultation with experts (*n* = 8) in several fields: Clinical (*n* = 3; professionals in: physiotherapy with a focus on burns and plastics, occupational therapy and clinical research director), HCI in healthcare (*n* = 4; professionals in: game development (*n* = 3) and digital health (*n* = 1)) and Psychology (*n* = 1; professional in: health psychology). The interventions were based on the observations of traditional physical exercises for the treatment of ULMI used in the hospital's out-patient clinics. Traditional physical exercises include upper body movements and exercises such as overhead, side-front and back arm raises and curls.

#### Study 1: pre-clinical

Five healthy children (males = 3 and females = 2), aged between 10 and 11 years (*M* = 10.6, *SD* = 0.52), from a local school were invited to use VR in a classroom after the end of the school day, with parental consent and their class teacher present. Children evaluated and gave feedback on the VR system’s usability using a questionnaire. Both VR-Archery and VR-Climbing tasks (described below) were performed by all the participants for 15 minutes each, in a counterbalanced design, to reduce order effects. No further instructions were given to the participants since the aim of this study was to inform the VR’s development by evaluating the system’s usability. All participants had normal or corrected to normal vision and no disability that could affect their performance of the exercise task. All participants had no clinical conditions or any kind of musculoskeletal disorders. No participants had a history of any mental health or neurological disorders and were not taking any medications that affect the central nervous system.

#### Study 2: clinical

Twenty-two participants (10 children with ULMI, 10 family members and 2 clinicians) were recruited. Ten children (male = 4, female = 6) with ULMI, with mean age 11.40 years (*SD* = 2.80, range = 9–16 years) participated to the study. All children were diagnosed with an ULMI (Burns sequelae (scar reconstruction arm) = 4; arm motor impairments = 2; elbow fracture = 2; multiple-exostosis = 1; nerve and muscle injury-head, arm, shoulder = 1). Inclusion criteria were ULMI for which the child was receiving rehabilitation from a psychotherapist or occupational therapist. The presence of injuries to the face or head that could hinder the correct positioning of the Head-Mounted Display (HMD) or pose an infection risk, along with learning or visual impairments or mental health issues that could affect the understanding and the performance of the rehabilitation, and a history of severe motion sickness or vertigo which posed a risk of nausea, epilepsy, disorientation, and anxiety to the children were exclusion criteria.

The two clinicians treating the children, a physiotherapist and an occupational therapist specialising in the field of burns and plastics surgery, plus ten family members (e.g. one parent for each child) who were present during the VR rehabilitation, also participated in the study.

Children with ULMI were invited to use VR with their usual clinician in a familiar room of the hospital. The VR intervention was described to them, and a five-minute tutorial was offered for the children to become familiar with the use of the VR system and the interactive devices. Once this process was completed, children were asked again whether they still wanted to proceed with the VR rehabilitation session. They were reassured that they had the choice to stop the session at any time. A consent form was signed, and the children were asked to put on the HMD. The main VR therapy session then began, taking around 30 minutes, 15 minutes per game. Once the VR rehabilitation session was completed, questionnaires and semi-structured interviews were conducted. The questions were asked taking into consideration the children's age. Where further explanation was needed by the child, the researcher offered clarifications, in keeping with good practice when working with children. Overall, each session lasted approximately an hour long.

### Materials

**System evaluation questionnaire** was carried out in both studies. To evaluate the VR system, a range of questions was used on a ten-point Likert scale (1 = not at all to 10 = very much). After the VR session, participants were asked a series of questions (e.g. “How much did you like the VR game”?) and they were asked to provide the level of difficulty and pain they perceived (e.g. “How easy or difficult did you find the VR game”?, “How painful did you find the rehabilitation process”?). Participants were also asked to evaluate the system’s usability and acceptability (e.g. “Was the system easy to use”?; “Would you like to play this VR game again”?). Perception of time was measured in minutes and seconds (e.g. “How long did it feel that you were into this virtual world”?).

**Observation notes** were taken during VR use in both studies by the HCI researcher located in the room with the participants. These observations aimed to record any interactions and behavioural responses towards the VR experience and identify design and deployment issues to help inform the VR design.

**Goniometer** device (Standard BASELINE® 12-inch plastic goniometer, (Model 12–1000—Fabrication Enterprises, Inc: White Plains, New York) was used by the clinical staff before and after the patient's rehabilitation to document the initial and subsequent range of motion, evaluate their progress and to determine the level of disability. We used the goniometer only in study 2, to assess the physical therapy effectiveness of the VR rehabilitation and to personalize the system’s range of motion for each patient. We examined the differences between a range of movements: upper limb flexion, upper limb extension, upper limb abduction, and upper limb adduction, before and after the VR rehabilitation.

**Semi-structured interviews** were conducted only in study 2, with the children, family members and clinical staff to reflect on their experience using VR concerning technology acceptance, emotional affect, usability and future deployment. Staff was interviewed separately from children and family members. In particular, the interviews focussed on four main areas: (1) attitudes towards the VR rehabilitation session (e.g. “What did you like/dislike about the VR session compared to the normal therapy session?”, “Compare with how it is usually when you are doing your exercises, how much did you enjoy doing them today, with the game?”); (2) perceived difficulty and pain levels (e.g. “Compared to normal therapy sessions, when you are doing your exercises, how easy was it to make the movements?”, “Compared with how it is usually when you are doing your exercises, how painful or uncomfortable was it to do them today?”); system usability/acceptability (e.g. “What is your overall impression regarding the VR rehabilitation?”); and future VR deployment (e.g. “In the future, could you see this kind of therapy as a form of rehabilitation?”).

### Apparatus

An Oculus Rift (Oculus 2019) VR HMD system was used to stream the audial and visual content. A set of Oculus Touch Controllers was used as the interactivity device. The system was paired to two sets of Oculus Sensors to capture the user’s physical position and movements and incorporate them into the virtual environment. The Oculus Rift tracks the head movement to present the correct virtual-world image to the eyes (LaValle et al. 2014) and it constantly analyses the user’s head movement to control the view. This results in completely natural interactions between the user and the virtual environment, creating high rates of the presence and immersion (Desai et al. 2014). The HMD used in the study had an adjustable head strap, and the combined weight of the HMD (470 grams) and controllers (169 grams/per controller) is 808 grams, which makes the system comfortable for young users.

The VR system was developed using the Unreal Engine (Unreal Engine 2019) and Oculus SDK (Oculus 2019). The 3D models were created in Autodesk 3DS Max (Autodesk 2019), Substance 3D Texturing Suite (Substance 2019) and Speedtree (Speedtree 2019). To create a sense of embodiment, virtual hands were developed to present the user’s hands and synchronize the movement in the virtual space, reflecting the movement of the users in the physical space. To offer a personalized experience and avoid any frustrations, for each type of virtual rehabilitation, the patient’s range of motion was measured by a goniometer device before to the training, and the data were imported by the physiotherapist into the system to capture the trajectory of the arms for each exercise.

The VR content was displayed on a laptop screen, mirroring the user’s real-time virtual interactions, allowing the researcher, the clinician, and the family member to silently observe the procedure. A dictaphone was used when interviewing the children with ULMI, the family members and the clinical staff.

### Design process and virtual environments

Four major design iterations, over nine months, were conducted to design and develop the VR system. Each iteration involved focus groups, interviews and/or evaluations with representative users (study 1) or clinicians to improve the design. Each evaluation lasted approximately two hours.

First, a demonstration was conducted, between three clinicians and one developer. During the demonstration, the general possibilities of VR rehabilitation were illustrated. Afterwards, clinicians demonstrated a ULMI rehabilitation process to the developers and the VR content was discussed. These consultations resulted in two types of virtual rehabilitation, designed to mimic the conventional treatment the children received at the hospital: 1) *Archery* based on behind-the-neck overhead press, using a quiver on the user's back to encourage bending their arm and firing the arrow to help with stretching exercises and 2) *Climbing* based on an overhead exercise that requires patients to raise their arms above their heads.

**Fig. 1:**
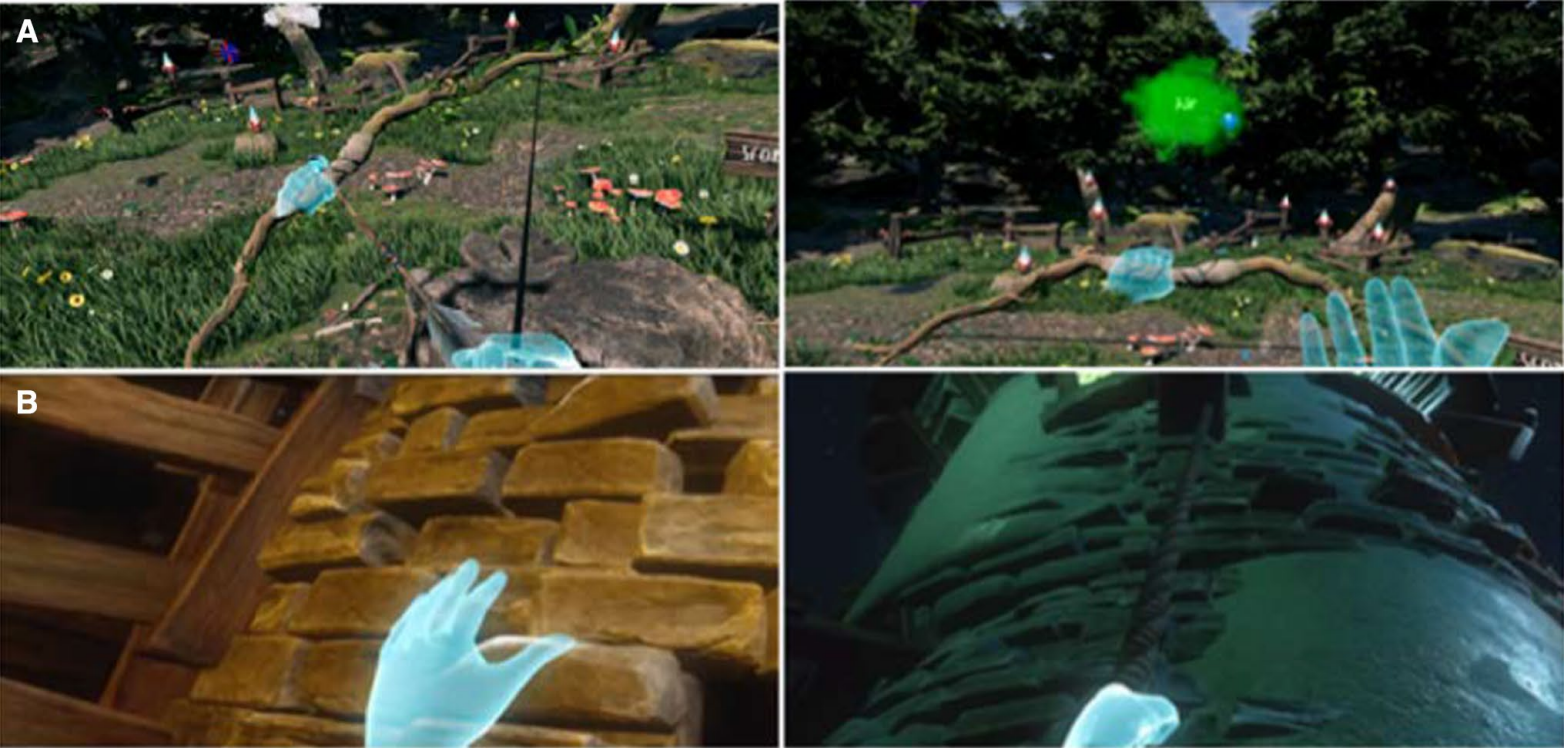
**a** VR-Archery: to the left, the arrow is in line with the bowstring to attach it. To the right, the arrow is released, and the target is fired. **b** VR-Climbing: to the left, the user is climbing via a VR-Climbing Wall. To the right via a VR rope

**VR-Archery**. A woodland environment with balloon targets and destructible gnomes was presented. The user was instructed to reach out with their non-injured arm to grab a bow that was floating in front of them and then to lift-up his/her injured arm, bending the elbow behind the back to grab an arrow from a quiver. Once the user reached the arrow s/he was instructed to bring the arrow in line with the bowstring to attach it. When it was attached to the bowstring the user had to hold the bow outstretched and pull back the injured arm holding the arrow. Then the user had to aim the arrow towards the target (i.e. balloon). The arrow was fired as soon as the finger was released. If the target was shot by the arrow, then the balloon popped, releasing colourful fairy dust into the air (Fig. 1a). If an arrow mistakenly shot another element (e.g. wood, ground), then the arrow would be stuck into the element and relevant sounds and visual effects would appear. To increase realism, the user was able to pluck the arrow from the wood and reuse it. Audio and visual feedback (e.g. blinking/pop sounds and fairy dust) were used to provide impact and encourage extra movement. To hit more distant targets, the arrow had to be pulled further back, indicated by a unique sound and visual effect on the arrow. To increase the gameplay time, we used a scoring system along with a score multiplier. This encouraged more advanced players to play faster while still receiving a challenge. The atmosphere of the scenario was light-hearted with playful background music and gnomes making high pitched sounds to increase children's interest and as a result to positively impact training tolerance and time. The task was repeatedly performed for 15 minutes.

**VR-Climbing.** An animated fairy appeared at the beginning of this scenario to indicate to the user where they needed to climb. Once the fairy scene was concluded the user was transported to the base of the tower. The tower wall consisted of 125 climbable bricks and 15 ropes. The user was instructed to climb up to the top of the tower while performing overhead arm raise exercises (see Fig. 1b).

To encourage the participants to follow the correct path that would maximise overhead exercise, the bricks were slightly highlighted with glowing, light-emitting algae. The user had to raise each arm alternately over the head keeping the elbows slightly bent. Once the brick was grabbed, the user had to lower the arm to climb up. In case the user failed to grab the brick, s/he would fall off the climbing wall. To prevent discouragement, checkpoints were developed in different levels for the user to land on, if they fell. As the path the user had to take was not pre-determined, the user needed to decide which path to take.

Once a dummy version of a VR children's rehabilitation system was created, the designer met the clinicians again to demonstrate the VR system. The clinicians suggested natural green environments for the archery and a reward-based scene for the successful completion of the climbing exercise, along with a scoreboard, cheering and applause sounds. A firework display was used to reward the climber on completion.

Following these consultations, a second version of the VR was developed and presented to clinicians. The final suggestions were to replace the fireworks in the climbing task with a calming night view since some of the patients could have burn injuries caused by fireworks. Following these amendments, five healthy children evaluated the VR rehabilitation system (study 1).

#### Study 1: pre-clinical testing

The results revealed positive attitudes towards the two rehabilitation scenarios. Even though children thought that the tasks were physically difficult to perform (VR-Archery: *M* = 7.2, *SD* = 1.35 and VR-Climbing: *M* = 5.8,* SD* = 1.92), most had high rates of enjoyment (VR-Archery: *M* = 7.0, *SD* = 2.35 and VR-Climbing: *M* = 9.4, *SD* = 0.55). VR-Archery and VR-Climbing were also found to alter the user’s time perception. Specifically, each participant spent 15 minutes in the VR environment but perceived the task duration to be significantly shorter than the real-time. For the VR-Archery, this was validated by a *t* test which compared the actual time spent in the VR and the time perceived by the user. The results revealed a significant difference of between the actual time spent in the VR and the time perceived by the user (*M* = 11.00 minutes, *SD* = 2.24); *t*(3) = 3.65, *p* = 0.035. The results were even greater during the VR-Climbing experience (*M* = 8.75, *SD* = 4.79) were users believed that they spend 50% less time than they did *t*(4) = 11.00, *p* = .000.

#### Design implication from study 1: pre-clinical testing.

Some minor usability issues were observed and reported, yet attitudes were positive, and users perceived low difficulty. VR-Archery issues were related to the correct archery moves. The initial design of the archery mechanics aimed to make it as realistic as possible, which required the user to line up the arrow to their eye height to determine the arrow trajectory. However, this technique required some prior experience of archery and therefore proved difficult for younger children. Only one user managed to perform the exercise correctly and mentioned during the administration of the questionnaire that they previously had archery lessons. Alterations were made to include a non-obtrusive targeting system that would indicate the arrow trajectory to make aiming easier. To balance the difficulty, the arrow had two firing states. When the bowstring was extended halfway then it would have low velocity, but the complete extension of the bowstring provided greater speed and accuracy. The difficulty concerns by the children prompted the development of a brief tutorial to help understand the gameplay mechanics.

The issues with the VR-Climbing related to the introductory scene, the visualization of the climbing path, the levels, checkpoints, and the movement distance. Specifically, the introductory scene began with a small climbing tower followed by an overhead rope linked to the main tower. This proved to be overwhelming so early in the scenario, and it was removed. In its place, a more relaxed introductory scene was developed, in which a fairy helped indicate to the user which path to take.

Multiple alterations were made to visualize the climbing path. Specifically, in the beginning, every climbable brick was glowing, pulsating or had a different colour. Participants suggested during use that these visual cues were distracting. Therefore, it was decided to have a small section of interactive brick highlighted to reveal the climbing path to the user. The climbable bricks' brightness was increased as the user came closer to grab them.

Finally, the biggest concern during the evaluation was the distance of the bricks from each other. The scenario was developed by adult males with large arm spans and even though designers aimed to reduce the distance while developing the prototype, some bricks remained too far apart, preventing some users from moving past certain points. The distance of every brick was considerably reduced to address this issue. Following the above alterations, the system was now ready to be deployed in clinical healthcare settings (study 2).

## Results (study 2: clinical)

### Data analysis

A range of quantitative and qualitative data sources were analyzed to assess the effectiveness of VR and how the technology was used to support ULMI rehabilitation. Descriptive statistics were used to examine the patient’s enjoyment, level of difficulty and pain and the system’s usability and acceptability. Means (*M*) and standard deviations (*SD*) are reported. For statistical tests, *p* = 0.05 was used to test significance. All statistical tests were carried out using the Statistical Package for the Social Sciences (SPSS) version 25.

To explore how VR was used by patients and perceived by consultees and clinicians, and to identify design challenges and opportunities, a qualitative content analysis of interview data was conducted, which revealed three core categories based on each participant group and four themes (Fig. 3). Interview data were anonymized, and participants referred to as Patient Number or Clinical Staff Number or Family Member Number. Page Numbers and Line Numbers were reported as well.

### System Evaluation Questionnaire

The VR system was found to be easy to use by all the children (10/10). Specifically, all the children with ULMI claimed that they would like to use the VR rehabilitation system to perform their rehabilitation sessions. The effectiveness of VR for ULMI rehabilitation was further corroborated by the reported low levels of pain (*M* = 2.85, *SD* = 0.29). Children with ULMI are generally dealing with painful rehabilitation. Specifically, participants claimed to have significantly higher rates of pain during conventional rehabilitation (*M* = 9.50, *SD* = 1.58) in comparison to the VR rehabilitation. The above statement was validated via a *t *test which compared perceived pain during the VR against the perceived pain of conventional rehabilitation. The results revealed a significant difference in VR rehabilitation and conventional rehabilitation (*t*(9) = 3.94, *p* = 0.003).

**Fig. 2:**
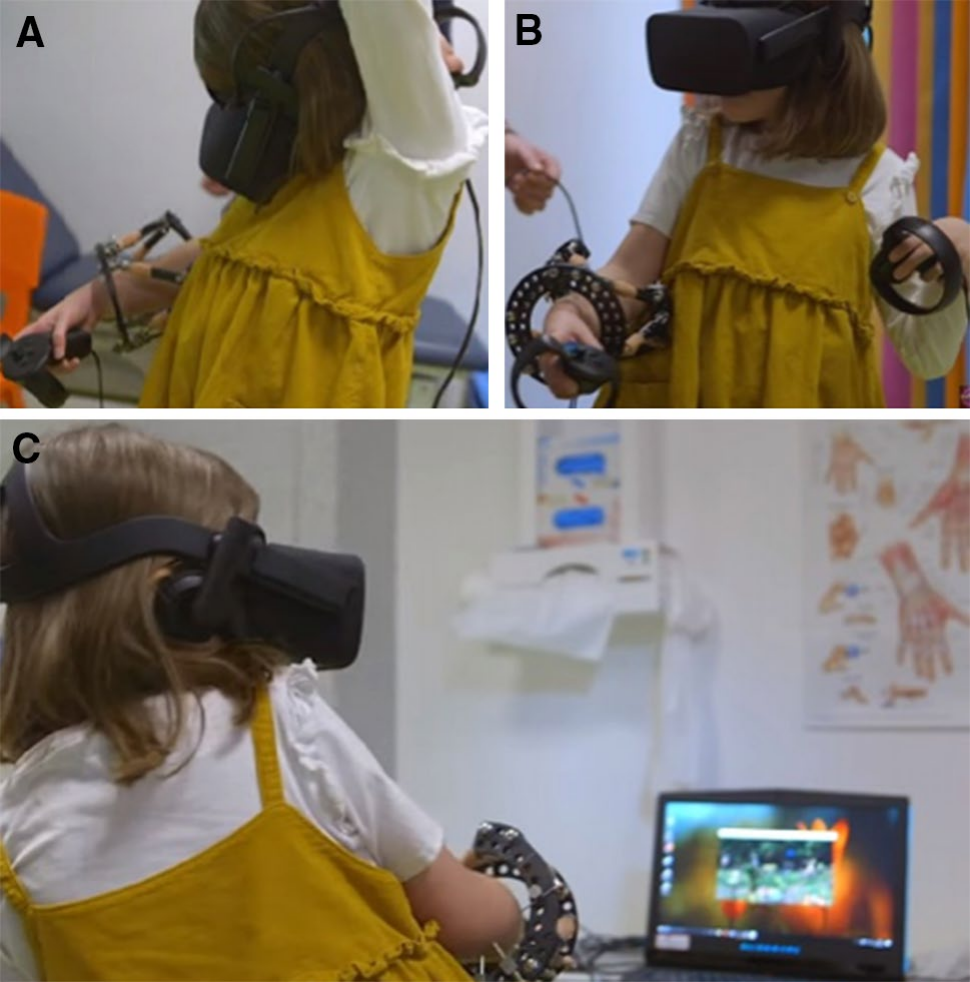
Multiple-exostosis patient using VR-Archery. The screenshot was taken from the BBC broadcasting of our study

It was also found that both VR rehabilitation games were effective in relation  the difficulty levels of the required movements. In particular, the rates provided for the VR-Archery (*M* = 3.20, *SD *= 2.74) and the VR-Climbing (*M* = 3.00, *SD* = 2.58) were relatively low. Interestingly, it was found that the VR-Climbing game which was reported to be slightly more challenging was also reported to be less difficult concerning the required exercise movements. Figure 2 depicts a patient suffering from multiple-exostosis performing a VR-Archery game. An additional *t* test analysis compared the levels of difficulty children with ULMI reported for the VR session (*M* = 3.00, *SD* = 2.58) compared to the conventional rehabilitation (*M* = 7.40, *SD* = 2.72). The results reported a significant difference in VR rehabilitation and conventional rehabilitation (*t*(9) = 3.67, *p* = .005).

The above findings were further corroborated by the high ratings given by the children with ULMI to each VR game (VR-Archery and VR-Climbing). In particular, the results indicated that all patients found the VR-Archery game easy to use, with clear and direct movements (*M* = 0.0, *SD* = 0.0), whereas the VR-Climbing was to some minor extent less usable (*M* = 0.7, *SD* = 0.48). The results revealed positive attitudes towards the VR-Archery game, with high reported rates of enjoyment (*M* = 9.00, *SD* = 0.94) and low reported levels of difficulty in usability (*M* = 4.15, *SD* = 2.03).

Similar results were also given for the VR-Climbing game, with slightly higher rates of enjoyment (*M* = 9.65, *SD* = 0.67) and difficulty (*M* = 5.05, *SD* = 2.14). Comparing the results, we can conclude that both VR rehabilitation games were perceived positively given the high ratings of enjoyment. However, the VR-Climbing game was slightly more challenging, and it was perceived by the patients as a little more enjoyable.

### Goniometer

Children's perceptions of the ease of their movement within the games were supported by data collected using a goniometer device. The device indicated significant differences in a range of movements: upper limb flexion, upper limb extension, upper limb abduction, upper limb adduction, after the VR. As presented in Table 1, the results suggest that VR rehabilitation can aid significant improvements in motion for children with ULMI.

**Table 1:** Goniometer data, *t* test results comparing the effectiveness of the upper limb VR pre and post ***p < .001; **p < .01; * < .05

	*n*	*M*		*SD*		*t*	*df*	*p*
		Pre	Post	Pre	Post			
Flexion	6	110.0	123.0	66.0	73.7	4.1	5	.01
Extension	5	64.0	68.0	16.4	5.7	8.8	4	.001
Abduction	4	66.3	84.3	31.5	7.2	4.2	3	.024
Adduction	4	81.3	83.8	8.5	12.5	19.0	3	.000

### Interviews

**Fig. 3:**
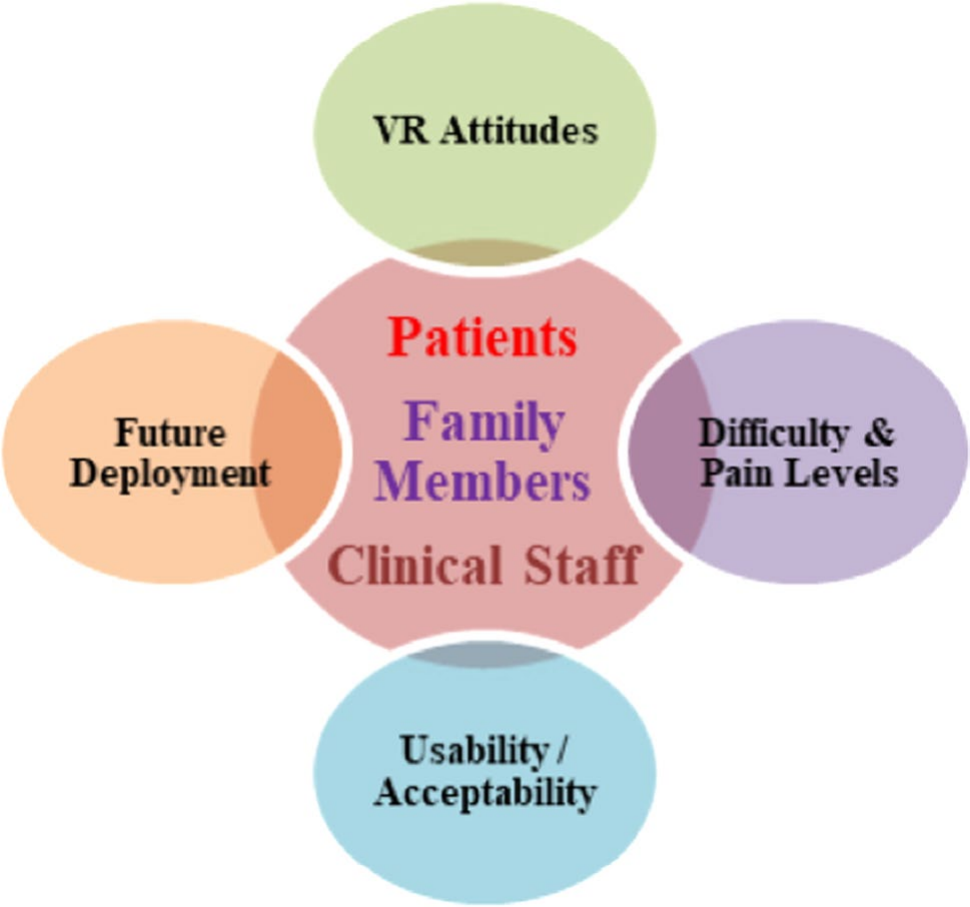
Patients (children with Upper Limb Motor Impairments), Family Members and Clinical Staff Themes

When assessing the potential of VR within children’s hospitals, it is essential to understand the unique aspects, advantages and limitations of the virtual experience, which make the VR technology viable and valuable. Data from all participants, including children with ULMI, their family members and clinical staff provided qualitative detail regarding “Attitudes towards the VR rehabilitation”, “Difficulty and pain levels”, “System Usability/Acceptability” and “Future VR deployment” (Fig. 3).

#### Attitudes towards the VR rehabilitation

Compared to the conventional therapeutic session, feedback from all children with ULMI (10/10) suggested that VR therapy was much more enjoyable than conventional therapy. This was further supported by most family members (8/10) and all the clinical staff (2/2). In particular, clinicians found VR to be an effective tool for children with ULMI (see Table 2).

**Table 2:** Content analysis interview data for the theme attitudes towards the VR Rehabilitation

Participant ID	Page Number	Line Number	Quote
Patient 4	2	23, 35–36	“The best bit was the feeling of actually being there […] It was more enjoyable because it felt like I had no injury, so it felt like I could just do it.”
Patient 5	1–2	23,16, 40–43	“It was really fun! […] Because you exercise but in a fun way, and like you can see loads of things around you. It’s definitely more different than the real world. And it’s kind of like fantasy, and I liked it so much”
Family Member 9	2	57–58	“He was 100 percent more engaged than usual […] I’ve never seen [children’s name] do his [rehabilitation] movements so happily, in many-many years”
Clinical Staff 1	1–2	20–26, 29–34	“I think they [children with ULMI] found the VR to be a very useful therapy tool […] they really enjoyed it. One child, in particular, got very excited about it and was very keen. She’s 10 years old. She has got a congenital condition and she’s got a frame on her arm. She’s quite compliant with her exercises. She does carry them out. But we found that during the VR, she perhaps hadn’t been doing her exercises in the past as much because she got quite tired from using, you know, from having to put her arm up like this. […] But with the VR she was doing it and overall, all children feedback was positive.”

#### Difficulty and pain levels

VR therapy was found to alleviate pain and reduce the level of difficulty for children with ULMI, according to all participant groups. Seven out of ten children with ULMI reported that during the VR rehabilitation, they did not feel any pain at all. While the rest of them (3/10) reported low levels of pain, discomfort and/or soreness. Similarly, seven out of ten family members and all the clinicians reported that no pain had been observed during the VR rehabilitation (see Table 3).

**Table 3:** Content analysis interview data for the theme difficulty and pain levels

Participant ID	Page Number	Line Number	Quote
Patient 2	3	101–105	“I felt zero pain! Because I was doing something at the same time, so I didn’t realise that I was doing them [referring to the rehabilitation exercises] […] I want another go! [Laughter]”
Patient 4	1–2	33, 38–39, 45	“Normally my shoulder hurts and feels heavy, but today I think it took me a bit longer until it started feeling heavy. For a while, I felt like I had no injury and that made me feel free”
Family Member 2	4	109–112	“She didn’t look as though she was in pain at all. She didn’t look as though she had any restrictions. She looked as though she could’ve done a lot more than she realises possible to do while she was just…”
Family Member 5	5	147–158	“I think because it is a game and she didn’t want to not win, I think she would just carry on through the pain. Whereas exercises [Conversation overlapped by the patient to agree with the Family Member’s statements]. But because it’s a game, she wants to go to the top or she wants to shoot that, no [Conversation overlapped by the patient to agree with the Family Member’s statements]”
Clinical Staff 2	4	130–136	“None of the kids have reported any pain during, even kids that would normally report pain with their exercises haven't reported any pain during [the VR rehabilitation]. Some of them have said their arms have been quite tired, or a bit achy because they've done more than they would normally, and their arms been up for longer, but no actual pain and discomfort at all.”
Clinical Staff 1	2	36–39	“We had a patient with a heavy circular metal frame with rods going through the bones. And from my point of view, that was quite interesting to see how much she tried. So, it was good for my point of view as a therapist for stamina to build up that tolerance of strength.”

#### System usability/acceptability

Six out of ten children with ULMI and seven out of ten family members reported positively on the system's usability, sharing that it was easy to use and that the children’s interactions appeared to be natural. The rest of the patient (3/10) found the system neither easy nor difficult, suggesting that the usability of the system was moderate, while one child with ULMI (1/10) found the system slightly challenging to perform the exercises effectively and efficiently while enjoying the experience. They also reported the positive effects it had on the perceived pain levels as well as the increased duration to exhaustion. Overall, the VR therapy was well received by the patients and the family members. Similar views (2/2) but with concerns regarding the stamina effects (1/2) were reported by the clinical staff (see Table 4).

**Table 4:** Content analysis interview data for the theme system usability/acceptability

Participant ID	Page Number	Line Number	Quote
Patient 10	3	91–92	“Compare to how usually I am doing the training [the VR rehabilitation session] was much easier to do”
Family Member 2	2	109–112	“It was just so fluent, she didn’t have to think about what she was doing. She was naturally doing the movements, and the exercises what she’s supposed to be exercising anyway. So, I think the fact that it distracts them at the same time could also be a good thing for the pain as well because it will help children to forget about the pain sometimes and just [the conversation was overlapped enthusiastically by the patient to validate that she didn’t felt pain]”
Family Member 8	2	60–62	“She was really enjoying it. And I think she was probably sustaining it longer than she was doing her exercises as well because it was obviously much less boring”
Clinical Staff 1	3–4	76–77, 117–124	“I think VR probably encouraged them to get more movement and move differently for a sustained period of time. […] I was concerned especially with [children’s Name] because she was so excited, and she wanted to do it that we had to say -Right, I think you need a rest now-. Because she would just keep going and going and going. And I was worried that later in the day, that would have perhaps detrimental effect that it would hurt too much.”

#### Future VR deployment

Finally, all patients (10/10) and all family members (10/10) and all clinical staff (2/2) suggested that they could see a future where VR could be deployed in real-world clinical settings to enhance the rehabilitation processes of children with ULMI (see Table 5).

**Table 5:** Content analysis interview data for the theme future VR deployment

Participant ID	Page Number	Line Number	Quote
Patient 10	5	180–182	“I would like to play this game again and if we could have this definitely in the future available, I think that would be really good for everybody”
Family Member 8	8	328–332	“I definitely feel that if this type of training [referring to VR rehabilitation] will be available in the future, it would be a useful addition to rehabilitation care. Because I think kids find their exercises really boring. And I think even if you tell them to do 10 min, they probably don’t know what 10 min is. And I think this does sustain the exercises for a lot longer”
Clinical Staff 1	16	652–658	“I would feel very positive if VR technology were gradually to come into the clinical areas and be used more widely and routinely in care of children in the future. […] I don’t think it replaces everything. But it certainly has a place. […] Yeah, I will be happy, yeah, for VR to be part of our therapy”

## Discussion

Children with ULMI often undergo painful and repetitive therapeutic processes to improve the functional abilities of the affected area. VR has the advantage of creating a distracting virtual experience which provides opportunities for the patient to reduce pain and enhance their rehabilitation (Matsangidou et al. 2017b). The purpose of this study was to explore whether VR is a feasible solution for children with ULMI who are dealing with a painful therapeutic process within a hospital environment. Using quantitative and qualitative approaches, we found that VR could be a successful solution for physical rehabilitation for children with ULMI. More importantly, our findings suggested that VR appeared to be very effective for this clinical population. It was found that VR could: (1) improve functional disabilities; (2) alleviate perceived pain; (3) reduce the perceived difficulty of rehabilitation exercises; (4) increase exercise duration; and (5) produce positive emotions towards the physical therapies.

Data collected via a goniometer device suggested that VR rehabilitation could aid a significant improvement in functional disabilities for children with ULMI. Significant improvements emerged in a range of upper limb movements such as flexion, extension, abduction, adduction after the VR rehabilitation. These preliminary findings are especially important for the research community since they validate the effectiveness of VR for children with ULMI.

Based on subjective reports of pain given by the children with ULMI, the findings revealed a significant reduction in participants' experience of pain. This was further supported by subjective reports which indicated lower pain levels during the VR session in comparison to conventional rehabilitation. The findings are in line with previous studies which suggested that VR can decrease the perception of pain in younger patients. A study of fifty-seven children demonstrated that pain from phlebotomy was significantly lower during the use of VR (Gold et al. 2005). VR for paediatric intravenous placement also proved to be an effective solution in a sample of 20 children who received an intravenous placement for magnetic resonance imaging/computed tomography (Gold et al. 2006). The study used VR Street Luge (Fifth Dimension Technologies, Irvine, CA), which was a racing game. The patient was instructed to race on a hill while lying on a skateboard. Positive results were also recorded in the use of VR on paediatric patients with acute burn injuries. This study (Das et al. 2005) examined the effectiveness of VR on the procedural pain of burn dressing changes. The research involved seven children playing a video game, in which the patient was able to shoot monsters with the use of a pointer. The results revealed the effectiveness of VR based on video games. Our findings are in line with previous studies and suggest that VR could be used as an alternative form of analgesia with minimal side effects and positive impact in the physical hospital environment.

The findings also revealed lower levels of the perceived difficulty in the rehabilitation exercise performance during the VR session in comparison ratings of conventional rehabilitation. The findings are in line with a study that suggested that VR can influence positively the perception of task difficulty during upper limb muscle contraction (Matsangidou et al. 2017a, b). To examine this, participants were asked to hold their Baseline Mass in an isometric contraction for as long as they could with their elbow at an angle of 90º flexion. Via VR technology, the participants’ attention was diverted from the painful sensory signal and that as a result, decreased the perception of task difficulty. The implications of minimizing the perceived task difficulty are substantial since patients will be able to perform further rehabilitation or to perform it more intensely. This will result in an improved willingness to engage in the rehabilitation for a longer period which has the potential to improve the physical outcomes of the rehabilitation.

Our findings suggest that VR technology can alter time perception in young children, to the effect that they feel less time has passed when using VR. These effects were in line with several previous clinical research studies, which suggested that VR can contribute to a reduction in the duration of a painful process (Schneider 2007; Schneider and Workman 2000; Schneider et al. 2004; 2011; Wiederhold and Wiederhold 2007). We believe that the results highlight the positive impact VR can have on children suffering from ULMI or other functional disabilities. These results indicated that VR can enhance the context of conventional rehabilitation, providing an enjoyable rehabilitation solution. It was shown that via VR rehabilitation the children were enjoying their therapy, doing much more in it, moving more freely, responding more positively and complying more readily with practitioners. Therefore, one can assume that VR rehabilitation can have a positive impact on patients' psychological and physiological wellbeing.

We also demonstrated positive emotions towards the use of VR for rehabilitation, reported by patients, clinical staff and family members. Previous research has shown that between patients and clinicians a therapeutic relationship developed which is a key factor for a good outcome in long-term care and this therapeutic relationship can be also implemented in the virtual environments (Gorini et al. 2007; 2008; Horvath and Symonds 1991; Horvath et al. 2011; Matsangidou et al. 2020; Martin et al. 2000; Norcross 2002). We observed that VR allowed children with ULMI to engage more positively and confidently in their physical rehabilitation. As a result, VR offered new experiences which transform the up to now tedious training into an enjoyable task for the children patients with ULMI and their therapists, promoting a positive therapeutic connection between them.

## Implications and recommendations

This feasibility study aimed to examine and consider potential research directions towards a more deployable VR system for clinical rehabilitation for children with ULMI. Considering the sensitive nature of this domain, we set out some directions for future deployment.

Throughout the study, we observed the importance of examining the clinicians' conventional rehabilitation structure as a vital factor that contributed to the VR design. We found that the design of a successful VR rehabilitation system must be based on the requirements of conventional interventions and that specific instructions should be given to the user to be able to correctly perform the rehabilitation task. For example, VR-Archery was selected because it imitated the movements of conventional upper limb rehabilitation. However, it was found that the tasks were not always performed correctly. To resolve this issue a targeting system that indicated the arrow trajectory was used. We encourage researchers and clinicians to present clear directions to the user to enhance the accurate task performance within the virtual environments.

We also found that a crucial factor for the interaction design to deliver meaningful experiences is to gain a sense of autonomy. Such autonomy can be exhibited by the ability to choose the climbing path direction. As aforementioned, the system used highlighted bricks for the user to climb up the wall executing the correct exercise. However, each user was able to take a different climbing path based on the climbing direction s/he chooses. These autonomies allowed the patient to freely experience the virtual environment, whilst being in a safe environment supported by guidelines developed by a clinical team. We encourage researchers and clinicians to capitalize on the sense of “guided” autonomy within the virtual environments.

Patient’s capabilities are a crucial factor to design patient-centred rehabilitation exercises. We found that customised scenarios based on the patient's range of movement are required to ensure that the exercise stimulates with the appropriate difficulty. Therefore, we encourage the design and research community to adjust the rehabilitation exercises based on each patient’s needs.

Another vital factor for the design is to imitate natural interactions to reduce confusion and increase the usability of the system. For example, a “hold and release” button which imitated the natural grabbing movement is a better solution than an “automatic hold”. This results in natural interactions between the user and the visual environment, which lead to high levels of autonomy and immersion.

Finally, when considering the VR content design is important to take into consideration the patients’ ability. For example, our initial design required participants to stretch their arm behind their back to grab an arrow from a virtual quiver. However, some patients’ impairments meant that they could not reach that far. A solution was devised that provided the clinician with the ability to alter the distance of the quiver so the participants could use it at any stage of their rehabilitation. Therefore, it is highly recommended that information is gathered from playtests at multiple stages to identify potential issues.

## Limitations and conclusions

This study examined the acceptability of the deployment of VR for children with ULMI, their clinicians and family members. The VR rehabilitation system of this study was run within a hospital environment to aid conventional rehabilitation. Even though very positive results were observed for this deployment of VR in healthcare, the study was limited to a relatively small sample. The small sample can be explained due to ethical restrictions of recruiting non-adult clinical patients. Nonetheless, the VR system was used for the treatment of a variety of ULMI, allowing us to observe different aspects of the interaction design and deployment considerations. It was explored using mixed methods and from a multidirectional perspective (patients, clinicians, family members) which enhanced the breadth and depth of understanding of VR's acceptability. Future studies should examine the use of VR for clinical rehabilitation in a large-scale sample; indeed, we are planning a multi-site study to this end.

An additional limitation of the study was that no goniometer data were collected from a traditional therapeutic session. We used the goniometer to assess the physical therapy effectiveness of the VR rehabilitation and to examine the differences between a range of movements. Future studies should use goniometer data to compare the VR’s rehabilitation effectiveness to the traditional therapeutic session. That being said, we suggest future studies to run comparisons between a traditional therapeutic session and VR. In addition, future studies should examine the role of immersion by comparing non-immersive, semi-immersive and fully-immersive VR systems.

Finally, we would like to note that contrary to the traditional therapeutic session, the VR rehabilitation session required the participant to perform the exercise tasks while holding an Oculus Rift controller. The controller weight approximately 165 g which might have caused some sort of discomfort to the patient. Future studies should add to the traditional therapeutic session a weight approximate to the controller’s weight.

The study contributes to the emerging body of research on the use of medical technology for children with physical motor impairments by presenting the opportunities VR offered to this patient group and the challenges we faced in the deployment of VR in this context. We believe this paper lays the foundations for the deployment of VR on a large scale in clinical environments and we can see a future where VR will be used as a personalized home-based rehabilitation solution.

## Data Availability

The approvals from the university and the National Health Services restrict us from sharing any raw data.
